# Brazilian private health system: history, scenarios, and trends

**DOI:** 10.1186/s12913-021-07376-2

**Published:** 2022-01-10

**Authors:** June Alisson Westarb Cruz, Maria Alexandra Viegas Cortez da Cunha, Thyago Proença de Moraes, Sandro Marques, Felipe Francisco Tuon, Arivelton Loeschke Gomide, Gisele de Paula Linhares

**Affiliations:** 1 Fundação Getúlio Vargas – EAESP, São Paulo, Brazil; 2 Pontifical Catholic University of Paraná, Curitiba, Brazil; 3 School of Business, Pontifical Catholic University of Paraná, Curitiba, Paraná 80215-901 Brazil; 4 Department of Otolaryngology, Head and Neck Surgery, School of Medicine, Stanford University, Palo Alto, USA; 5 Pontifical Business School at the Catholic University of Paraná – EUA, Curitiba, Brazil

**Keywords:** Private health, Brazil, Market concentration

## Abstract

**Background:**

Health care is a complex economic and social system, which combines market elements and public and social interest. This combination in Brazil, like systems in China and United States of America, is operationalized through the public and private system. The sector represents approximately 9% of the country’s GDP, of which 56% is privately sourced and 44% is of public origin. In the private sector includes a structure with 711 private health institutions, 47 million beneficiaries and revenues of US$30 billion a year.

**Methods:**

Therefore, this research describes and analyzes the complementarity of Private Health before the Brazilian Unified Health System, highlighting its main characteristics, scenarios, and trends in the face of the health system and the Brazilian market. This descriptive and exploratory research uses secondary data from various sources, submitted to quantitative data analysis methods. The object of the research is the history of private health in Brazil and its main actors.

**Results:**

The data are organized into three groups, each with its approach of collection and analysis. Thus, it is perceived as the notorious growth of large operators, to the detriment of operators with a lower concentration of beneficiaries; the increasing concentration of the market through mergers and acquisitions promoted by large publicly traded corporations, especially in regions with a lower rate of private health coverage; and the growth of the sector through business plans, whose central characteristic is the dependence on the country’s employability rate.

**Conclusions:**

It is possible to perceive an intense trend of concentration of Brazilian private health in large institutions that have capitalized and have a great appetite for growth through mergers and acquisitions, whether from smaller operators or health institutions that integrate their health networks, following complementary health models already consolidated in countries such as China, and the United States of America, among others. This concentration projects a market with fewer options and competitiveness, reduction in transaction costs and increase the operational effectiveness of health care.

**Supplementary Information:**

The online version contains supplementary material available at 10.1186/s12913-021-07376-2.

## Introduction

Brazil is a country of continental dimensions, with a territory of 8.5 million square kilometers and a population of 211 million people, being the sixth most populous country on earth [1]. Its health system comprises the state’s performance through the Unified Health System, and the private initiative [2].

Since the 1990s, many efforts have been devoted to health care in Brazil by public or private means. Although the efforts are commendable, the country presents many challenges around health [3] owing to the intense socioeconomic inequality present in the country [3–5]. This can be verified by the degree of income concentration, also known as a measure of inequality, the Gini index of 50.9 [1, 4], a value measurement of countries such as Zambia (57.1) [6] and Zimbabwe (50.3) [6].

Like the challenges of the Canadian [7] and United States of America systems [8], the Brazilian health system is formed by a complex and challenging network of health service providers and buyers [9], all with intense challenges of promoting an adequate cost-effectiveness ratio in health [10, 11].

With the public and private actors, the Brazilian health system is divided into two sectors: public and private. The public sector comprises state funding; the private sector is financed by public and private resources, mostly for profit, and comprises different modalities of insurance and private health plans [12]. Table 1 describes the main characteristics of each of the members of the health sector in Brazil.

**Table 1:** Overview of the health system in Brazil

Description	Public	Private
System	Unified Health System	Private Health
Main Regulatory Body	Ministry of Health	National Agency for Supplementary Health
Funder	Union, States and Municipalities	Individuals and Legal Entities of a Private Nature
Service Provider	Public and Private Entities	Private Entities
Year Regulation	1988	1998
Coverage	Universal	Consumers

Historically, private health systems have been stimulated by a series of government policies, either through the accreditation of services, and the remuneration and creation of hospital units among others [13].

The health sector in Brazil represents approximately 9% of the GDP [4, 14], of which 56% has a private and 44% has a public origin [4, 15]. The health sector employs 4,418,871 [14] people and comprises a structure with 711 private health institutions [16], 256 dental plan operators [16] and 6642 hospital units [17] among others.

The Unified Health System, created from the Federal Constitution of 1988 [4], is based on the principle of health as a citizen’s right and duty of the state1. Currently, approximately 75.5% of the Brazilian population is served solely and exclusively by the Public System [18], which, despite its historical achievement in scope and access, suffers strongly owing to chronic underfunding [4, 5].

The other 24.5% of the population have access to health through private health [16], which is strongly linked to the care of people through individual or family contracts (19%), business (68%) and collective (13%) [16].

Private performance in Brazilian health has intensified since 1964, after the military coup, when a series of reforms drove the expansion of the private health system. Since then, a series of historical events have fostered private performance in the health sector, leading to a significant expansion of the provision of health services through private health [12], as described in Table 2 below:

**Table 2:** Historical series of relevant private health events in Brazil

Period	Event Description
1500–1822	Creation of hospital structures - Santas Casas [19]
1897	Creation of the General Directorate of Public Health [20]
1889–1930	Start of health care and social security system [20]
1933–1938	Extension of social security to most workers in urban areas [21]
1953	Creation of Ministry of Health [12]
1950–1960	Beginning of the first medical entities providing services financed by companies, with service predominantly focused on industrial workers [22]
1964	Initial development of private health companies (Decree-Law 200) [22]
1964	Expansion of hospital structures [12]
1964–1988	Crisis in the health system and social security Expansion of the health system by private means [12, 23]
1988	Decentralization of the Health System [24]
1990	Creation of the Unified Health System (Law 8080 and 8142) [4]
1996	Creation of the Provisional Contribution on Financial Transactions [24]
1998	Regulation of private health plans [24]
1999	Creation of the National Health Surveillance Agency [24]
1999	Beginning of private equity practice in private health companies [25]
2000	Creation of the National Agency for Supplementary Health (Law 9961) [24]
2000	Definition of health financing responsibilities - Constitutional Amendment 29 [24]
2001	Psychiatric Reform Law [24]
2004	Start of capital opening of Brazilian health companies [25]
2006	Pact for Health [24]
2006	Creation of the National Primary Care Policy and the National Health Promotion Policy [24]
2008	Creation of 24-h Emergency Care Units [24]
2011	Creation of Private Plan Operators Program - ANS Resolution 277 [16]
2019	Minimum Governance Practices - ANS Resolution 443 [16]

Currently, private health is regulated and supervised by several government and organized civil society institutions and forums such as the Supplementary Health Council [26], National Supplementary Health Agency [16] and Supplementary Health Chamber [27]. Its operation takes place through private health institutions, which are assigned to manage, market, and provide health plans, with the purpose of medical, hospital and dental care to their beneficiaries [26].

As of December 2020, there were 47,631,224 private health users [16], assisted by 711 hospital medical operators [16], with revenues of 30.4 billion (US$). Table 3, some of the main data of the sector and its respective representation in the Brazilian context.

**Table 3:** General data on the representativeness of Private Health in Brazil

Description	2010	2020
Health Spending (% of GDP) [4, 14]	8.3	9
Proportion spent on Private Health (%) [4, 15]	54.2	56.07
Proportion spent on Public Health (%) [4, 15]	45.8	43.93
Coverage Rate on Private Health Plans (%) [16]	22.3	24.5
The Hospital Medical Organization (Unit) [16]	1045	711
The Dental Organization (Unit) [16]	374	256
Assets of Private Health Entities (US$ - Billions) [26]	$ 11,764.71	$ 21,323.53^b^
Private Health Programs (Users) [16]	44,937,350^a^	47,615,162
U-files of individual or family plans (Users) [16]	9,560,381^a^	9,043,414
Users of Business Plans (Users) [16]	28,877,931^a^	32,192,328
U-kind Collective Plans (Users) [16]	6,643,512^a^	6,308,420
Users of Unidentified Plans (Users) [16]	943,990^a^	71,000
And Direct Jobs in Health (People) [14]	–	4,418,871
And Direct Jobs in the Private Health Sector (People) [14]	–	3,429,759
And Direct Jobs in the Public Health Sector (People) [14]	–	989,112
Revenue from Private Operators Payouts (US$) [16]	$ 15,592,201,463.79	$ 30,498,100,687.32
Operator Assistance Expenses (US$) [16]	$ 12,658,972,366.36	$ 22,090,892,703.13
Operator Administrative Expenses (US$) [16]	$ 2,427,299,367.83	$ 2,886,992,082.35
Operators’ Business Expenses (US$) [16]	$ 504,765,502.02	$ 961,252,886.21
Hospital Structures (Units) [17]	6907	6642
Hospital Beds Brazil (Unit) [17]	435,793	404,770
Private Hospital Beds (Unit) [17]	295,463	254,982
Beds per Thousand Inhabitants (Thousand Inhabitants) [17, 28]	2.23	1.91

^a^2011

^b^2016

In view of the initial presentation of the sector and the notorious importance of Private Health in the Brazilian context, the objectives, methods, results, discussions, and conclusions of the research are presented below.

## Objectives

The main objective of this study is to understand the general and specific context of Brazilian private health, its scenarios, and trends, with emphasis on the analysis of market concentration and recent processes of mergers and acquisitions.

## Methods

### Study design and technical aspects of research

This research is described as descriptive and exploratory, with the use of secondary data from various sources submitted to quantitative data analysis methods. The object of the research is the history of private health in Brazil, as well as its main actors. The data are organized into three groups, each with its approach to collection and analysis, as shown in Table 4 below:

**Table 4:** General framework of research methods

Group	Source data	Data Type	Form of Analysis
Historical and regulatory documents	Sites and reference searches	Documents and laws	Descriptive documentary analysis
Industry Data	Industry data repository sites	quantitative	Quantitative data analysis
Market	M&A Data Repository Sites	Quantitative documents and data	Descriptive analysis and network analysis

The first group of “Historical and Regulatory Documents” plays an important role in the research, as it allows the identification and analysis of the relevance and history of the private health sector in the Brazilian context.

The second group called “Sector Data” presents a descriptive statistical analysis and explains the historical series of evolution of the sector, as well as the measurement of the indices of market concentration IHH - Herfindahl-Hirschman (1) and RC5 - Concentration Ratio of the five largest [29] role players, adapted to the private health sector, according to equations below:

1 $$\mathrm{IHH}=\sum \mathrm{ni}=1\left(\mathrm{Carrier}\ \mathrm{Beneficiaries}\ \mathrm{Amount}/\mathrm{Total}\ \mathrm{Private}\ \mathrm{Health}\ \mathrm{Beneficiaries}\right)$$

2 $$\mathrm{RC}5=\sum 5\mathrm{i}=1\left(\mathrm{Beneficiary}\ \mathrm{Amount}\ \mathrm{of}\ \mathrm{the}\ \mathrm{Five}\ \mathrm{Largest}\ \mathrm{Volume}/\mathrm{Total}\ \mathrm{Private}\ \mathrm{Health}\ \mathrm{Beneficiaries}\right)$$

The resulting analysis of the IHH and RC5 assume values between 0 (no market concentration) and 1 (total market concentration). For analysis and interpretation, the scale of the credit market analysis was adapted, where estimates between 0.10 and 0.18 represent moderate concentration and, above 0.18, high market concentration.

The third group, “market”, presents an analysis of relational networks through the Software Ucinet [30], version 7.724. From the elaboration of the adjacency matrix, the analysis of the patterns of interactions of processes of division, incorporation, and mergers between entities of health legal entities was elaborated, with *graph theory analysis based* on the identification of private health institutions that appear as buyers or sellers, in the period from 2018 to 2020. This analysis methodology uses graphs to be analyzed descriptively and square or rectangular matrices, also known as socio matrices (*X*). The matrices allow the visualization of relationships and patterns that would hardly be perceived in the sociograms of points and lines. In the matrices, *the rows (g)* represent the sent links, while the columns *(h)* represent the received links or *(j).* The links sent and received have important implications for the calculation of local and global centrality degrees and in the identification of subgroups in the network. The notation for representation of a socio matrix can be expressed in (3).

3 $$\mathrm{X}-\mathrm{g}\ \mathrm{x}\ \mathrm{h}$$

The data sources for identifying the operations of mergers and acquisitions, from data mining on sites specializing in them totaling 196 sources can be consulted through Additional file 1.

## Results

**Fig. 1:**
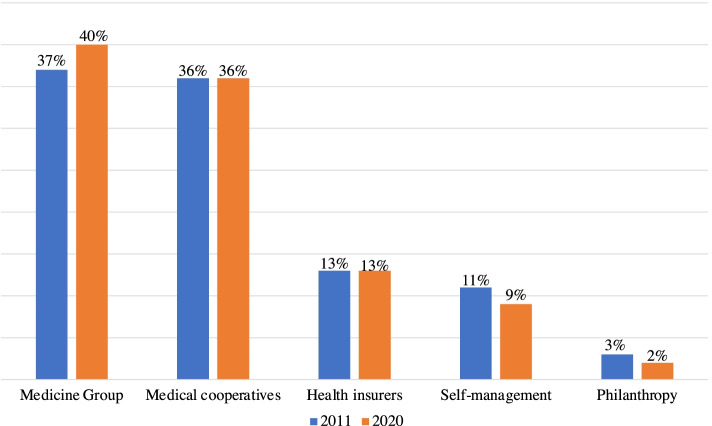
Distribution of Private Health Plans (2011–2020) [16]

The private health operate through more than one corporate typology of a legal entity, called “modalities” [16]: group medicine (40%), medical cooperatives (36%), health insurers (13%), self-management (9%) and philanthropy (2%). These organizations establish contracts for the provision of health services with their beneficiaries, protecting their users from the direct cost linked to the risk of falling ill and observing the principle of mutualism [2]. Figure 1 the modalities in the last 10 years:

Private health operators relate to their beneficiaries through contracts, with a predominance of contracts, called health plans, linked to companies through the so-called “collective business” plans, a fact that is repeated in all regions of the country. Interestingly, this type of contract is related to the level of employability of the country; therefore, it suffers more severe oscillations in a volatile economic system such as the Brazilian system. Table 5 contract modalities and their geographical distribution in the country regions.

**Table 5:** Distribution Types of Private Health Plans by Regions in Brazil 2020 [18]

Type of hiring	North	Northeast	Southeast	South	Central West	Unidentified	Total
Corporate collective	1,128,874	4,001,832	20,128,894	4,604,834	2,314,606	28,261	32,207,301
Individual or family	391,840	1,755,343	5,084,947	1,264,072	537,884	4299	9,038,385
Collective by adhering	235,715	820,018	3,767,746	1,021,740	464,381	4701	6,314,301
Uninformed	3064	10,354	48,928	4663	3799	1	70,809
Unidentified collective	0	77	338	13	0	0	428
Total	1,759,493	6,587,624	29,030,853	6,895,322	3,320,670	37,262	47,631,224

Regarding the size of private health institutions, there was a significant reduction in their number, from 1045 institutions in 2011 to 711 in 2020 [16]. This evidence is confirmed by analyzing the data in Table 6, which shows a growth of 29% in the number of beneficiaries linked to operators that have more than 500,000 beneficiaries, with a decrease in all other groups, whose intensity of decrease is in smaller institutions, reaching 57% decrease in institutions with 2001 to 5000 beneficiaries.

**Table 6:** Grouping of beneficiaries and Horizontal Analysis (2011–2020) [16]

Grouping	2011	2020	HA
Over 500,000 beneficiaries	17,600,739	22,715,394	29%
100,001 to 500,000 beneficiaries	12,276,731	11,704,100	−5%
50,001 to 100,000 beneficiaries	6,410,403	5,669,947	−12%
20,001 to 50,000 beneficiaries	5,567,096	4,682,160	−16%
10,001 to 20,000 beneficiaries	2,482,548	1,761,421	−29%
5001 to 10,000 beneficiaries	1,080,166	757,635	−30%
2001 to 5000 beneficiaries	451,840	271,783	−40%
1001 to 2000 beneficiaries	110,730	47,105	−57%
101 to 1000 beneficiaries	44,755	21,370	−52%
1 to 100 beneficiaries	806	309	−62%

Table 7 shows an intense increase in the last 10 years in the general concentration of the private health market, from an RC5 index of 0.22 in 2011 to 0.29 in 2020, reinforcing the hypothesis of an increase in market concentration, which is intense, following the trends of countries such as China [31] and the United States of America [32].

**Table 7:** Historical Series Beneficiaries and RC5 (2011–2020)

Year	Total Benefit [16]	RC5
2011	46,025,814	0.22
2012	47,846,092	0.23
2013	49,491,826	0.24
2014	50,531,748	0.27
2015	49,279,085	0.27
2016	47,648,903	0.27
2017	47,111,682	0.27
2018	47,121,811	0.28
2019	47,058,415	0.28
2020	47,631,224	0.29

**Fig. 2:**
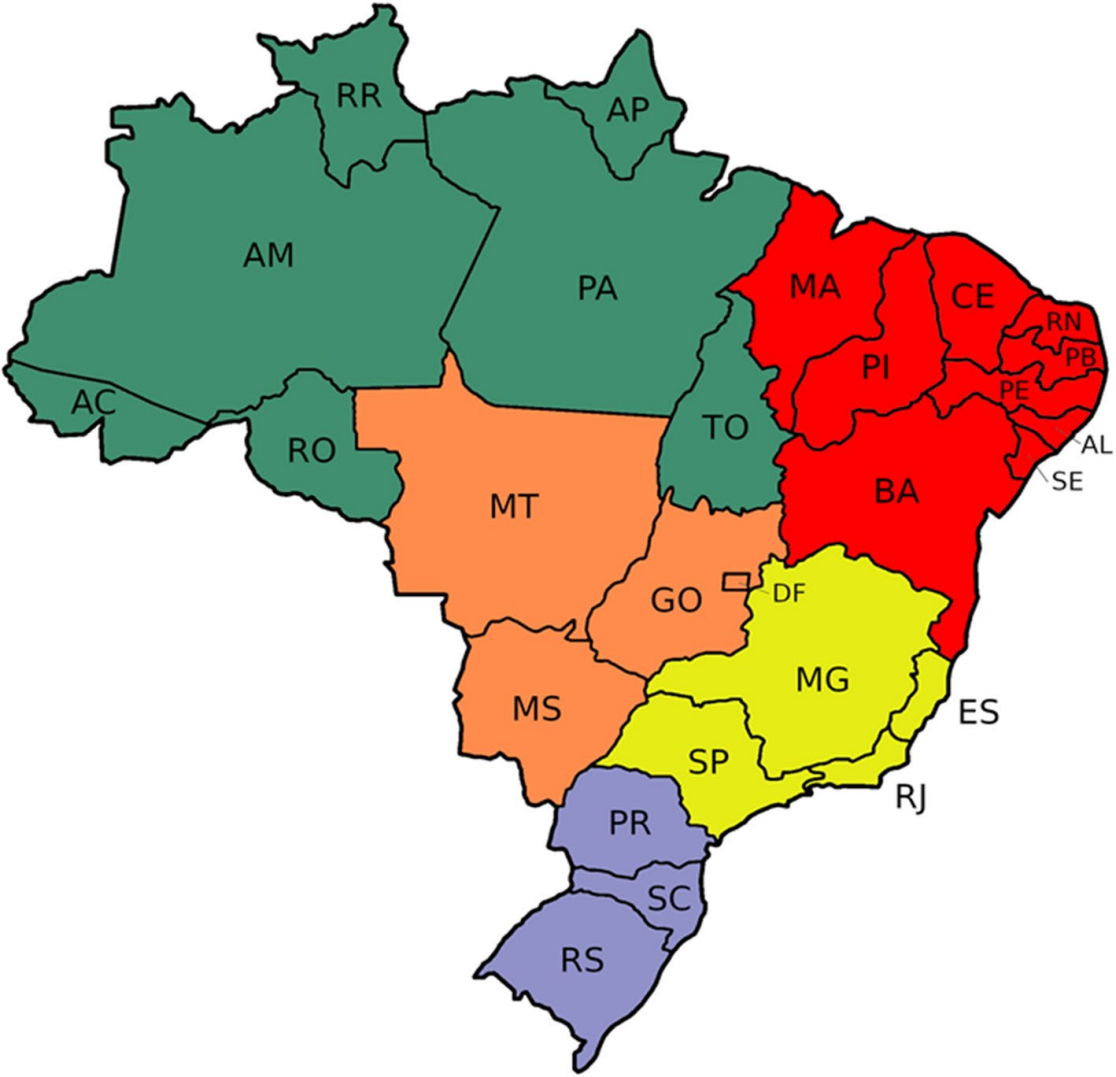
Private Health Coverage and RC5 by Brazilian Region (2011–2020)

By analyzing the RC5 index of the regions of Brazil, it is possible to understand that the continental dimensions of the country raise extremely different realities, although all demonstrate the increase in market concentration if we compare the years 2011 and 2020. The north and northeast regions of the country show an intense market concentration (0.58), with indicators that exceed the scale of 0.50, that is, more than half of the beneficiaries are concentrated in the largest five operators of these regions. The southern region has greater market dispersion, in addition to the lower variability in the period (2011–2020). Its RC5 index had a result of 0.26 in 2011 and 0.27 in 2020, as shown in Fig. 2.

Table 8 the 10 largest institutions operating in the Brazilian supplementary health system, as well as the resulting HHI in 2011 and 2020. The stability of the institution called Bradesco Saúde (0.07) is perceived in the leadership of the IHH, with intense growth in the period of analysis of the publicly traded company called Notre Dame Intermédica Health (0.07). Two institutions deserve special attention when analyzing the indicator of horizontal analysis, Hapvida Medical Care (0.06) with growth of 140% and São Francisco Health Systems (0.02) with growth of 425% in the analysis period.

**Table 8:** Ten Largest Private Health Institutions by Beneficiaries (2011–2020)

Private Health Institutions	Beneficiaries Quantity 20,112	IHH 2011	Beneficiaries Quantity 20,202	IHH 2020	HA
Bradesco Health S.A.	2,988,834	0.07	3,277,018	0.07	10%
Notre Dame Intermédica Health S.A.	2,140,143	0.05	3,241,622	0.07	51%
Amil International Medical	2,624,621	0.06	2,893,453	0.06	10%
Hapvida Medical Care	1,134,584	0.03	2,721,072	0.06	140%
South America Cia Health Insurance	1,279,444	0.03	1,858,761	0.04	45%
Unimed National Central	1,168,769	0.03	1,808,907	0.04	55%
Unimed - Belo Horizonte	971,061	0.02	1,297,348	0.03	34%
San Francisco Systems and Health	146,728	0.00	770,029	0.02	425%
Unimed-Rio Cooperativa Médica	774,619	0.02	736,615	0.02	−5%
Caixa de Assist. dos Funcionários	693,620	0.02	634,214	0.01	−9%

The increase in beneficiaries and market concentration can take two main forms: ordinary growth, or mergers and acquisitions. In the Brazilian private health market, like the United States of America [33] and Costa Rica [34], mergers and acquisitions have increasingly presented itself as alternatives. In the healthcare market, health insurance operators have been especially active in buying and selling assets. Of the 196 transactions of mergers and acquisitions of the Brazilian health market carried out between 2018 and 2020, 91 private health institutions are buyers of assets (53) or sellers (38).

Table 9 the predominance of acquisitions by private health institutions, focused on other private health plan (27) or hospitals (18), thus promoting market concentration and service delivery through their hospitals.

**Table 9:** Assets acquired by Private Health Institutions (2018–2020)

Assets Acquired by Supplementary Health Operators	Number of Operations
Private Health Institutions	27
Hospitals	18
Benefits Administrator	3
Miscellaneous (Clinics, Brokers, Laboratories and Technology Companies)	4

According to Table 10 private health institutions were predominantly sold to other private health institutions (27), with few events of selling operators to hospitals (6).

**Table 10:** Private Health Institutions sold (2018–2020)

Typology of Buyer Entities	Number of Operations
Private Health Institutions	27
Hospitals	6
Diagnosis	2
Miscellaneous (Clinics, Brokers, Laboratories and Technology Companies)	3

**Fig. 3:**
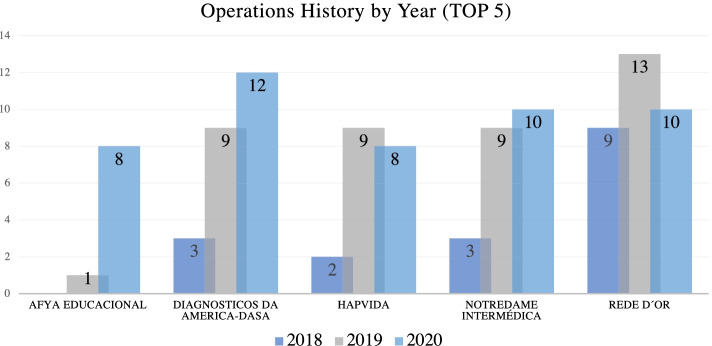
Main buyer entities (2018–2020)

According to Fig. 3 among the five main health entity asset buyers in the periods 2018 to 2020, two are private health institutions. Hapvida Health Care and Notre Dame Intermédica with 19 and 22 operations respectively, stand out as they both are publicly traded in the Brazilian market.

**Fig. 4:**
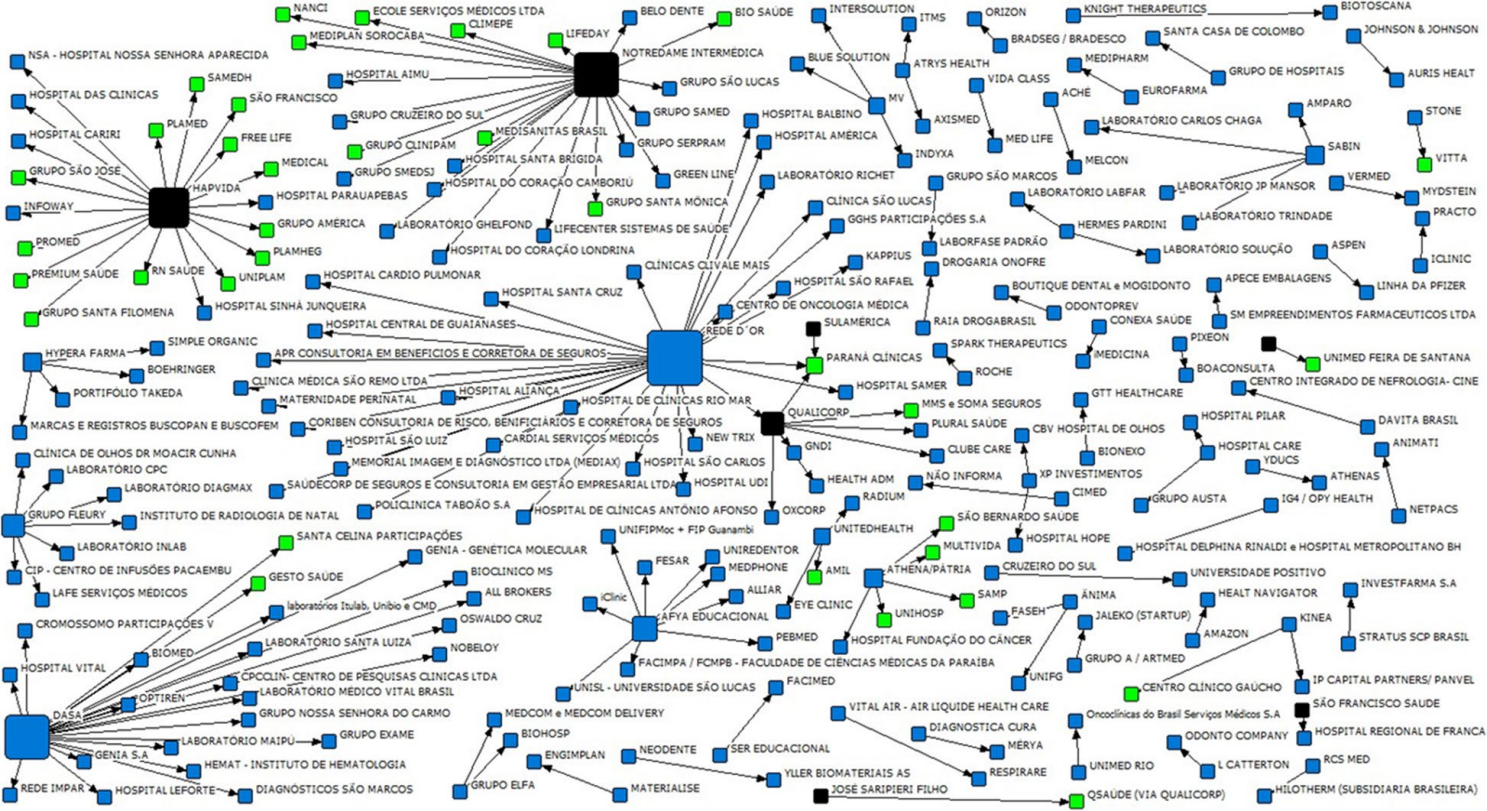
Sociogram of mergers and acquisitions in the health sector (2018–2020)

By observing the totality of mergers and acquisitions in the health sector in Brazil, from 2018 to 2020, as shown Fig. 4 centrality of operators that appear as buyers of assets in the market (black), which represent a relevant growth of their operations through mergers and acquisitions, a variable that helps in the analysis of the quantitative decrease of active operators in the market. However, the operators sold in the period present themselves in green, with a relational link with their buyer, represented by the black arrow.

As shown in Table 11 some operators are especially prominent in mergers and acquisitions in the period demonstrated. Among them, there is the institution Notredame Intermédica, being the third institution with the highest degree of total centrality in the private health market (22.0), followed by Hapvida in fourth place (19.0), and Qualicorp in seventh place (7.0), all publicly trading on the Brazilian stock exchange.

**Table 11:** Main buyer entities (2018–2020)

n.	Name of The Institution	Degree of Centrality	Operation Category
1	Rede D’Or	30.0	Hospital
2	Dasa	23.0	Diagnosis
3	Notredame Intermédica	22.0	Private Health Institutions
4	Hapvida	19.0	Private Health Institutions
5	Afya Educational	9.0	Medical Education
6	Fleury Group	7.0	Diagnosis
7	Qualicorp	7.0	Private Health Institutions
8	Athena/Homeland	5.0	Hospital
9	Hypera Pharma	4.0	Pharmaceutical
10	Sabin	4.0	Diagnosis

Table 12 shows the main characteristics of the 10 main private health institutions in Brazil, which together concentrate more than 19 million beneficiaries [16].

**Table 12:** General data of the 10 largest private health institutions in Brazil (2020)

Operator	Users	IHH	AH (2011–2020)	Mergers and Acquisitions (18–20)	Accident Rate	IDSS (2019) [35]
Bradesco Health S.A. (1984) [36]	3,277,018	0.07	10%	1.0	76.45%	0.70
Notre Dame Intermédica S.A. (1968) [37]	3,241,622	0.07	51%	22.0	71.40%	0.94
Amil Assist. International Medical. (1978) [38]	2,893,453	0.06	10%	0	77.06%	0.91
Hapvida Medical Care. (1991) [39]	2,721,072	0.06	140%	19.0	66.50%	0.75
South America Health Insurance Company. (1895) [40]	1,858,761	0.04	45%	1.0	76.90%	0.76
Unimed National Central. (1998) [41]	1,808,907	0.04	55%	1.0	82.80%	0.93
Unimed - Belo Horizonte. (1971) [42]	1,297,348	0.03	34%	0	68.89%	0.94
San Francisco Health Systems. (2019) [43]	770,029	0.02	425%	1.0	–	0.86
Unimed-Rio Medical Cooperative. (1972) [44]	736,615	0.02	− 5%	1.0	72.00%	–
Caixa de Assist. dos Funcionários (1944) [45]	634,214	0.01	− 9%	0	77.50%	–

The obvious concentration of the market in fewer operators and the absence of change in the overall number of beneficiaries creates “giants” in the market. Among them, the market leader in 2020, with an HHI of 0.07 and growth of 10% in the period 2011 to 2020 is Bradesco Health, whose growth strategy is strongly focused on common shares, making little use of growth via mergers and acquisitions in the years 2018 to 2020, with a centrality degree of only 1.0.

Although Bradesco Health leadership is solid throughout the period 2011–2020, the massive mergers and acquisitions operations of the operators Notre Dame Intermédica and Hapvida Health Care, both publicly trading on the Brazilian stock exchange, have been demonstrating relevant results and contributing intensely to the market concentration. Its HHI of 0.07 and 0.06 and centrality level of 22.0 and 19.0 respectively, demonstrate their appetites for growth support through mergers and acquisitions that focused on other private health institutions and hospitals.

By more careful analyses, some of the data from the 10 mains private health institutions in the country can be evidenced as different strategies of growth and market positioning in their corporate structures, volume of beneficiaries, average billing ticket, spending structure, value of their assets, HHI, degree of centrality, loss, and supplementary health performance index. Although the numbers are impressive, market interaction strategies are different, which will lead us to futile different performances in future individual analyses, with an apparent and inevitable growth of mergers and acquisitions operations.

Finally, it is worth highlighting the particularity within Brazilian private health of the cooperative system called Unimeds, founded in 1975, as one of the largest health cooperatives in the world [46]. It has 270 private health institutions with a total of 17,707,733 beneficiaries. Together they have an HHI of 0.37, representing the highest concentration of the Brazilian market with a growth of only 3% of the number of beneficiaries in the period from 2011 to 2020.

## Discussion and conclusion

Health care is a complex economic and social system [47], which combines market elements of public and social interest in a single environment. This intriguing combination in Brazil, like the Chinese [8, 31] and United States of America [32] systems, is presented through the public and private system, with the great challenge of providing access and health care for all Brazilian citizens.

The representativeness of health in the face of human existence and care and the economy are notorious, as perceived in the Canadian territory [7]. However, in Brazil, the challenges in the search for alternatives that promote a problem-effective discussion are emerging and endowed with many vulnerabilities. As the representativeness of the sector, before the economy, is on the scale of 9% of the GDP, employing more than 4 million people, the investment in intelligence from the previous history is urgent, either by access and quality of health care or by economic importance.

In this sense, describing and analyzing the complementarity of Private Health before the Unified Health System in history can help guide health scenarios and trends in Brazil. Based on this contribution, this research objectively and clearly demonstrates the main historical assumptions of Brazilian private health, enabling the essential perception of complementarity between the public and private health care systems.

This provocation currently directly interferes with 24.5% of the country’s health demand, which makes about $30 billion a year. Of this, approximately 74% is reinvested in spending on their health care through a decreasing number of institutions (711), with historical stability of total beneficiaries, increasingly converted to business plans (70%), this being the only modality in full growth in the last 10 years of the market. Importantly, the dependence of this type of contract, the performance of employability of the country, and the maintenance of contracts depend directly on the capacity of the country to generate employment and income.

Notoriously, the growth of large private health institutions, with a concentration of more than 500,000 beneficiaries, show an increase of 29% in the years 2011 to 2020, to the detriment of institutions with a lower concentration of beneficiaries, which present as percentage degrowth in any scale in this period. The market concentration in large institutions can also be evidenced by the exponential increase of the RC5 indicator from 2011 (0.22) to 2020 (0.29).

This reality is more pronounced in the regions of the country with the lowest rate of private health coverage, reaching 0.58 in 2020 in the north (9% private health coverage) and northeast (11% private health coverage) and is less accentuated in the southern region (0.27), whose private health coverage is 23%.

Therefore, it is possible to perceive an intense trend of concentration of Brazilian private health in large institutions that capitalized on and have a great appetite for growth through mergers and acquisitions, whether from smaller private health institutions that integrate their health care networks, following complementary health models already consolidated in countries such as China [8, 31], and the United States of America [32], among others.

This concentration projects a market with fewer options and competitiveness that can lead to a concentration of risks, raising potential frequencies of isolated failures according to user experience. However, according to health operators, they lead to a decrease in transaction costs and increase the operational effectiveness of care [11]. These hypotheses are still fragile in the literature applied to the private health sector and, therefore, they figure only as one of the most varied scenarios to be considered. Another important factor to be considered is the analysis of this scenario in countries of continental dimensions such as Brazil, which may present specific particularities concerning health, whether public or private, in its different regions, this favors exponentially the trend of market concentration growth, by an even greater flow of M&A operations, favored by the growing number of entities in the sector, listed on the stock exchange.

Finally, similar to the United States of America model [32], the complementarity of Brazil’s understanding of private health is worth highlighting, as it contributes to the access and qualification of health care, safeguarding premises of cost-effectiveness, quality, humanization and access to health, emphasizing the role of regulatory agencies in the sector, in the improvement of governance tools that guarantee the rights and duties of all stakeholders [48] from an integrated view of health, avoiding its eminently mercantility [33].

## Supplementary Information

**Additional file 1.**

## Data Availability

The data sets generated and / or course during the current study are available in the files sent to the journal.
